# The Liver

**Published:** 1890-06

**Authors:** 


### THE LIVER.

The liver is the largest gland of the body ; it is located at the right, above the lower border of the ribs ; reaching over to the left side, and lying close to the heart, overlapping the stomach.

It carries on several separate and distinct lines of work. We are best acquainted with its work of making bile, which is largely, if not entirely, made up of waste elements taken from the blood. Bile is a golden yellowish fluid ; but when vomited from the stomach it is green, and many people suppose that this is its natural color. The change is due to the action of the gastric juice.

It is an excretory and digestive fluid, converting fats which are not acted upon in the stomach, but in the small intestine, into an emulsion.

Bile also acts upon the mucous surfaces stimulating the absorption of food after it is digested.

The digestive fluid of the stomach is acid, the bile is alkaline, and the two being poured into the small intestine simultaneously with the food, prevent an irritating action of the gastric juice. Pure gastric juice is so strong an acid that it will irritate the hand if it is placed upon it. Were not the stomach protected in a peculiar manner, it would itself be digested with the food, as often happens in cases of sudden death after a person has just eaten a hearty meal.

Bile stimulates peristaltic action, by which means the food is moved along through the entire length of the intestines.

It is an antiseptic, and preserves the food from fermentation and decay in its slow progress through the twenty-five feet of alimentary canal. This is one of its most important uses. With the food is always taken a quantity of germs, and they would surely induce fermentation but for this preventive agent. About fourteen hours are required for the complete process of digestion from the time food enters the stomach until it is entirely absorbed.

If a single one of these several offices be absent or interfered with, it seriously affects the health of the individual.

The liver itself is a digestive agent. All the digested food, with the exception of a small portion of the fat, is absorbed and carried by the portal vein to the liver. The heart pumps blood direct into the general circulation, but all of it which goes to the stomach, spleen, and other abdominal viscera, is carried to the liver before it is allowed to go into the general circulation. Thus the liver acts as a filter for the blood received through the portal circulation, and completes the work of digestion. The stomach is only the ante-chamber where the process of digestion is commenced. There are twenty-five feet of intestines, and the work of these upon the food is vastly more important than that of the stomach. When the stomach and intestines have done all that they can do to the food, its nourishment is expended, and it is carried off as waste matter by the kidneys. The liver acts upon all its elements, which no other organ does. Starch constitutes one-half to two-thirds of all our food. When we take it into the mouth, the saliva begins to change it into sugar. It furnishes heat for the body, and muscular and brain force, hence we need a large amount of it. In the process of digestion, a large amount of sugar is manufactured. If all this were poured at once into the circulation, it would thicken the blood and render it so sluggish that the blood corpuscles would all be destroyed, and it would have to be hurried out of the system to save the person's life, so the liver goes immediately to work to change the sugar back into liver starch, and stores it up in its tissues. Then it begins slowly to change the starch back into sugar again, and doles it out for use as needed for force, heat, etc.

The liver is also a sort of rendering establishment. It takes what would otherwise be offensive and dangerous elements, and utilizes them, just as the rendering establishments of large cities take the dead animals and offensive garbage and make them of value. The liver takes all the broken down tissues, the millions of dead blood corpuscles, and works them over and changes them into material of which corpuscles can be made. The coloring matter found in these corpuscles is worked over into material which furnishes coloring matter for the eyes and hair, as well as furnishing color to the bile. Nature shows wonderful economy in thus taking old, worn out material, and converting it into such a variety of new uses.

The bile containing waste alkaline substances, also makes a little soap out of the fat, that aids in preparing the rest of the fatty material for absorption.

